# The Umbilical Cord Creatine Flux and Time Course of Human Milk Creatine across Lactation

**DOI:** 10.3390/nu16030345

**Published:** 2024-01-24

**Authors:** Walter Alexander Mihatsch, Bernd Stahl, Ulrike Braun

**Affiliations:** 1 Department of Pediatrics, Ulm University, 89075 Ulm, Germany; 2 Department of Health Management, Neu-Ulm University of Applied Sciences, 89231 Neu-Ulm, Germany; 3 Danone Nutricia Research, 3584 CT Utrecht, The Netherlands; bernd.stahl@danone.com; 4 Department of Chemical Biology & Drug Discovery, Utrecht Institute for Pharmaceutical Sciences, Utrecht University, 3584 CT Utrecht, The Netherlands; 5 Alzchem Trostberg GmbH, 83308 Trostberg, Germany; ulrike.braun@gelita.com

**Keywords:** breast milk, human milk, colostrum, creatine, umbilical flux

## Abstract

(1) Background: The aim of the present paper was to study fetal and infant creatine (Cr) supply to improve nutrition and neuroprotection in term and especially in preterm infants. The primary outcomes were the placental Cr flux at the end of pregnancy and the time course of human milk (HM) Cr. (2) Methods: The estimation of placental Cr flux was based on umbilical arterial and venous cord blood Cr in 10 term infants after elective caesarian section. HM Cr, creatinine (Crn), and macronutrients were measured longitudinally in 10 mothers across the first 6 months of breastfeeding. (3) Results: At the end of pregnancy, the mean fetal Cr flux was negative (−2.07 mmol/min). HM Cr was highest in colostrum, decreased significantly within the first 2 weeks of breastfeeding (*p* < 0.05), and did not change significantly thereafter. HM Cr was not correlated with HM Crn or macronutrient composition. (4) Conclusions: The present data suggest that fetal endogenous Cr synthesis covers the needs at the end of pregnancy. However, high colostrum Cr and HM Cr levels, independent of macronutrient composition, suggest that there may be a critical Cr demand immediately after birth that needs to be covered by enteral supply.

## 1. Introduction

Creatine (Cr, α-methyl guanidine-acetic acid) is a bioactive compound of human milk [1,2]. It is essential for cellular energy metabolism in order to replenish ADP to ATP via the creatine kinase (CK) reaction. In humans, the vast majority of Cr (95%) is maintained in the muscle cells, including cardiomyocytes [3,4,5]. Some of the remainder is located in the brain and may be influenced by dietary intake in children [6,7]. The main organ affected by a deficiency in Cr is the brain because it is essential for neural development [2,8]. Any errors in Cr synthesis or metabolism lead to neurological disorders, such as mental disabilities with autistic behavior. The newborn child receives his or her dietary Cr intake from human milk (HM) [2]. However, HM provides only approximately 9% of the demand of this compound. The remainder of the demand for Cr is met by de novo synthesis [9].

Human omnivore adults obtain up to half of their daily needs from eating meat and animal products, while the other half is synthesized de novo in the kidneys and liver and transported to the target organs via the bloodstream [10]. In adults, the plasma and tissue Cr levels depend on dietary habits such as vegetarianism [8,11,12], and vegetarians have lower blood and muscle creatine levels than omnivores. However, no information is available on the influence of diet regime on human milk Cr. A constant daily rate of approximately 2% of the Cr pool is lost via spontaneous degradation to creatinine (Crn) and has to be restored [13,14].

Creatine deficiency syndromes (CDS) represent a group of inborn errors of metabolism [8]. Three inherited defects in the two-step biosynthesis and transport of Cr have been described [8]. Cr synthesis requires the amino acids arginine, glycine, and methionine. These biosynthetic defects include deficiencies in the L-arginine-glycine amidinotransferase (AGAT) enzymes necessary to produce guanidinoacetic acid (GAA, step one) and guanidinoacetate methyltransferase (GAMT) for creatine synthesis from GAA (step two). The third is a functional defect involving the creatine transporter (SLC6A8) [15,16], impairing Cr transport and uptake into the target cell. Clinical features include severe mental disability, autism, movement disorders, hypotonia, speech delay, and early-onset epilepsy [8]. CDS symptoms have been successfully ameliorated using dietary supplements such as creatine monohydrate (Cr·H_2_O) [6,17].

Cr has a high bioavailability and is readily absorbed from both ingested meat and dietary supplementation with creatine monohydrate via the small intestine [18]. While the effect of dietary Cr and the contribution of de novo synthesis have been studied extensively in adults, much less is known about neonatal and especially fetal or preterm infants’ Cr supply and metabolism. A cross-sectional study found Cr concentrations of 60–100 μmol/L in pooled human milk > 15 days of lactation, and higher values within the first two weeks of breastfeeding [19]. These findings were reconfirmed in a second study. HM Cr decreased significantly in colostrum from days 0–5 to d14 and d28 in term infant mothers and a similar trend was observed in preterm infants [20]. However, opposite findings, namely, an increase in Cr in pooled samples of mature HM from month 1 to months 2–3, were found in an individual mother [21].

It is unknown whether there is the active synthesis or transport of Cr in the human breast or the passive diffusion of Cr from plasma into human milk. In a cross-sectional study, similar concentrations of Cr (60–70 μmol/L) were found in the breast milk and maternal plasma of 20 omnivorous mothers, with no change from 1–2 to 5–6 weeks of breastfeeding, consistent with passive diffusion [9]. However, in several studies [19,20,21], HM Cr was higher than creatinine (Crn) in contrast to plasma reference values [22], suggesting active Cr enrichment in HM.

The majority of the available data on human milk Cr are limited by the fact that Cr and Crn were not measured longitudinally across the entire breastfeeding period of at least six months. It is also unknown whether the Cr concentration is constant during a single breastfeed.

In growing rat pups, similar to human newborns, only 12% of the total Cr pool was obtained from milk, whereas the great bulk of Cr was accreted via de novo synthesis [23]. However, during fetal life, at least in the spiny mouse, there appears to be a limited capacity for endogenous Cr synthesis until approximately 90% of pregnancy has passed. Maternal Cr, transferred across the placenta, may be essential until the Cr synthesis and transport system matures in preparation for birth [24]. If these results also apply to humans, extreme premature infants may be at risk of early-life Cr deficiency [24] and, in fact, Cr deficiency has been suggested in very preterm infants based on urinary GAA, Cr, and Crn levels [25]. Human placental Cr metabolism has recently been reviewed [26]. In brief, the human placenta contains Cr and is able to produce and transport Cr. Placental Cr content and Cr transporter mRNA expression sharply increased in fetal growth restriction compared to controls, without affecting maternal plasma Cr or venous cord blood Cr. Several preclinical studies have suggested that Cr is a promising neuroprotective intervention for hypoxic ischemic injury [26,27,28,29,30]. However, fetal and preterm infant Cr metabolism are largely unknown [26].

The present study was designed to obtain more information on infant and fetal Cr supply to further improve nutrition and neuroprotection in term and especially in preterm infants.

Therefore, the aims of the present study were as follows:

- To estimate the placental Cr flux at the end of pregnancy by measuring umbilical arterial and venous cord blood Cr concentrations immediately postpartum after caesarian section.
- To analyze whether the Cr concentration in breast milk is constant during a single breast meal (intraindividual variability).
- To analyze the time course of the association between HM Cr and HM Crn, fat, carbohydrates, and especially protein. It is unknown whether Cr is independently secreted into HM or whether it just parallels, e.g., HM protein or Crn concentration.
- To analyze human breast milk Cr concentration longitudinally, starting with colostrum throughout the first six months of breastfeeding, to study if colostrum has higher Cr levels, suggesting increased immediate postnatal needs.

## 2. Materials and Methods

### 2.1. Umbilical Cord Blood Samples

Twelve expecting healthy mothers scheduled for an elective caesarean section were recruited from the obstetric department after obtaining informed consent. The inclusion criteria were a healthy pregnancy without complications, no gestational diabetes or any diseases, and carrying a healthy term fetus (gestational age > 37 weeks). The exclusion criteria included intestinal malformations, malformations potentially affecting food intake, and chromosomal abnormalities.

After the caesarean section, 4–10 mL of arterial and venous blood each was extracted postnatally from the placental end of the umbilical cord. The placenta and cord were discarded in accordance with local guidelines. Plasma was isolated from the blood samples via centrifugation and immediately stored at −20 °C until analysis. The day after birth, the participants were again approached on the maternity ward and the purpose of the study and its aims were discussed a second time. After written informed consent was obtained, the plasma samples were used for the study. Past medical history and dietary preference (omnivore or vegetarian) were recorded.

### 2.2. Collection of Breast Milk

Intra- and interindividual variability in breast milk creatine.

To assess the effect of the timing of breast milk sampling, the intra- and interindividual variability in human milk Cr and Crn were analyzed in seven healthy omnivorous mothers who were exclusively breastfeeding, on demand, healthy, term infants between two and ten months of age. All participants were recruited from the hospital’s outpatient department. Pumping breast milk was a well-known home routine procedure to all participants for private organizational reasons and spare frozen breast milk for substitution was available from all participants. Written informed consent was obtained. In all participants, about 30 min before the next routine breastfeeding, a single full breast feed from one breast divided into 6 mL aliquots was obtained using an electronic pump (Symphony Breast Pump, Medela Medizintechnik, 85386 Dietersheim, Germany). From five to fifteen samples were obtained from each participant.

### 2.3. Longitudinal Study on Breast Milk Creatine in Omnivore Mothers

Twelve mothers who intended to breastfeed were recruited from the hospital’s maternity or pediatric wards. The inclusion and exclusion criteria were defined as already mentioned. The purpose of the study was explained to both parents. A home freezer (−18 to −20 °C) had to be available. After written informed consent, the mother was enrolled into the study and her medical history, including dietary preferences, food supplements, and medications, was recorded. The attending midwife assigned to weekly home visits with the family was also informed. During their time in hospital, the mothers received professional lactation consulting. For the collection of breast milk samples, the mothers were asked at regular time points to obtain 10–15 mL of early-feed breast milk and store it in the freezer compartment of their home fridge at −20 °C. The postnatal timepoints to obtain breast milk samples were as follows: 1st week (colostrum), 2nd week (transitional milk), weeks 3–4 (mature milk), 2 months, 3 months, 4 months, and 6 months. All samples were obtained using identical breast milk pumps (Medela Symphony, Medela Medizintechnik GmbH & Co. KG, 85386 Dietersheim, Germany), which were provided for the study. The frozen samples were picked up from the mothers’ homes and transported to our facilities for a batch analysis every two weeks. A temperature of −20 °C was maintained during transport as well as storage.

Mothers were excluded if they became ill within the first two weeks after delivery and were unable to breastfeed or if the first-week sample was missing. Mothers were not excluded if they became ill later in the course of the study and missed, at the most, one sample collection. If a participant wished to withdraw consent, she could leave the study at any time without further questions or discussion. Written agreements were to be made with the parents about what would happen to the samples that had already been collected. Samples had to be discarded at the request of the parents. Excluded or withdrawn participants had to be replaced by new volunteers to maintain the target cohort size of ten. Urinary Cr was not analyzed.

### 2.4. Preparation and Analysis of Blood Samples

The Cr concentrations were determined using the Barrit reaction, where Cr is coupled to diacetyl, yielding a red-colored compound which is stabilized with 1-naphthol. Absorbance measurements were performed at 546 nm [31,32]. The limit of detection was 0.05 mg/dL.

Creatinine (Crn) was quantified using the Jaffe method [33] without deproteinization and rate-blanked with compensation on a Roche Modular system (Roche Diagnostics, Mannheim, Germany). The limit of detection was 0.06 mg/dL. Both Cr and Crn measurements were performed by an accredited laboratory (Medizinisches Labor Bremen, Bremen, Germany).

The fetal cardiac output amounts to approximately 400 mL/kg/min, of which 21% are transferred to the placenta towards the end of pregnancy, with an umbilical cord flow volume of 82 mL/kg/min [34]. Based on these values, the mean fetal Cr supply was calculated according to the following equation:

(1) $$Fetal\;Cr\;Supply=\sum_{i=1}^{10}\left(\frac{{CrV}_{i-}\;CrA_{i}}{10}\;x\;82\;\frac{mL}{kg\;x\;min}\right)$$

where *CrV_i_* and *CrA_i_* represent the Cr concentrations in mg/mL in the umbilical cord vein and artery, respectively, for child *i*.

### 2.5. Preparation and Analysis of Human Milk Samples

Proteins were precipitated with hydrochloric acid. Then, the sample was centrifuged and the supernatant was filtered through a 0.45 µm membrane filter. The sample was then neutralized with sodium hydroxide solution and again filtered through a 0.45 µm membrane filter.

Reference samples of Cr and creatinine were purchased from Sigma-Aldrich (Merck KGaA, Darmstadt, Germany). For the Cr analysis, ion chromatography with a gradient pump and variable wavelength detector (Dionex DX500; Dionex Corp., Sunnyvale, CA, USA) was used. The Hypersensil Hypercarb column (4.6 × 100 mm) was obtained from Thermo Fischer Scientific (P/N 35007-104630) and the pre-columns Aminopac PA1 5 × 50 mm and Aminopac PA1 4 × 250 were obtained from Dionex (P/N 37022 and P/N 37023). Deionized and vacuum degassed water was used as an eluent. After injecting 50 µL, the separation was performed with a flow of 1.0 mL/min at a temperature of 30 °C. Cr was then detected at 200 nm. Our previous unpublished data have shown that there is no loss of Cr within 6 months of milk storage at −34 °C.

The creatinine content was determined using a high-performance liquid chromatograph with a variable wavelength detector. The two Hypercarb columns, 7 µm × 100 mm and 4.6 × 100 mm, were connected in series and were obtained from Thermo Fisher Scientific (P/N 35007-104630). The mobile phase consisted of 22 mL of isopropanol, 1 mL of NaOH solution (1 mol/L), and 977 mL of water. In total, 50 µL of the filtered milk was injected and separated with a flow of 1.0 mL/min at a temperature of 30 °C. The detector was set to 235 nm.

HM protein, lactose, and fat concentrations were measured using mid-infrared spectrometry. Mid infrared analyses of milk were performed in accordance with ISO guideline ISO 9622:2013 [x]. Thawed human milk samples were homogenized by the gentle inversion of the sample container to avoid foaming. An aliquot of 1 mL was subjected to mid-infrared analysis using (Miris HMA™, MIRIS Human Milk Analyzer, MIRIS AB, 73523 Uppsala, Sweden, Miris HMA™ User Manual (www.MirisSolutions.com, accessed on 1 July 2023)). Each sample was analyzed in duplicate. Mid-infrared spectrometry analyzes the macronutrients of protein, fat, lactose, and total solids simultaneously. The calculation of digestible energy was based on these macronutrient contents [35,36]. The within-subject correlation between the analyzed creatine to protein ratio and the study timepoint was calculated.

### 2.6. Statistics

Cr concentrations are given as µmol/L. All data were computerized using Microsoft Excel (Version 16.81, Microsoft Deutschland GmbH, Munich, Germany) by a research nurse and double cross-checked by two of the investigators (UB and WAM). Statistical analyses were performed using SPSS (Version 9.9.0.0, IBM SPSS statistics) and RStudio (2023.06.1+524, PBC, Boston, MA, USA URL http://www.rstudio.com, accessed on 7 July 2023). Repeated measures correlations (rmcorr) were employed in conjunction with bootstrapped confidence intervals to analyze the association between the creatine (mg/L) to protein (g/100 mL) ratio and the postnatal timepoint. This statistical technique was specifically designed to examine the (common within-subject) association between paired measures collected from the same individuals on multiple occasions [37]. A significant difference was defined by a *p* value of less than 5%. There was no correction such as Bonferroni for multiple testing. The study was part of our infant nutrition research program, which started in 2010 and was approved by the institutional ethics committee (3 August 2010, S310/2010).

## 3. Results

### 3.1. Umbilical Creatine Flux

**Figure 1:**
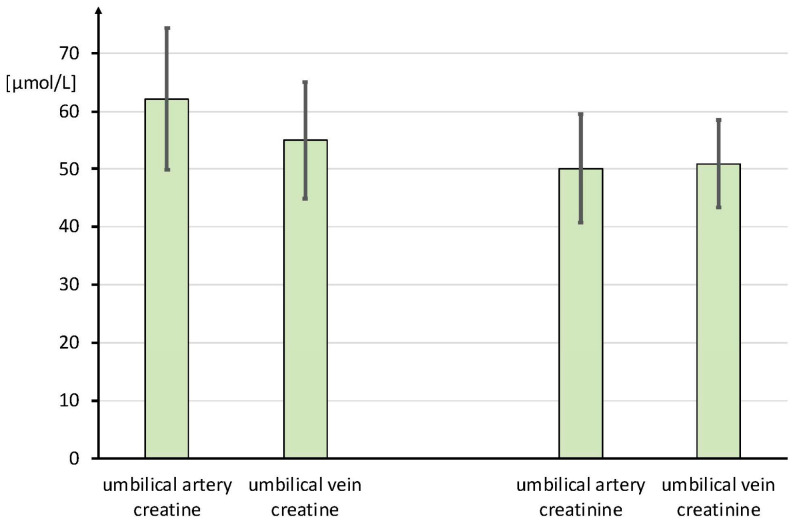
Creatine and creatinine concentrations in the umbilical artery and vein given in µmol/L. Data are given as mean ± SD.

Umbilical arterial and venous data were available in ten out of the twelve participants. Two participants had to be excluded for low umbilical arterial blood sample volume. The Cr concentrations were within the reference range for healthy newborns [38]. The mean Cr concentration was significantly (*p* < 0.05) higher in the umbilical artery than in the umbilical vein (65.0 ± 13.5 µmol/L vs. 57.5 ± 11.1 µmol/L, *p* = 0.017), suggesting an average negative fetal Cr flux of 2.07 mmol/min at the end of pregnancy (Figure 1). The arterial and venous Crn values were virtually identical (50.1 ± 9.4 and 50.9 ± 7.5 µmol/L respectively) and within the newborn infant reference range (67.3 ± 11.5 µmol/L) [39].

### 3.2. Intra- and Interindividual Variability in Breast Milk Creatine and Creatinine

**Figure 2:**
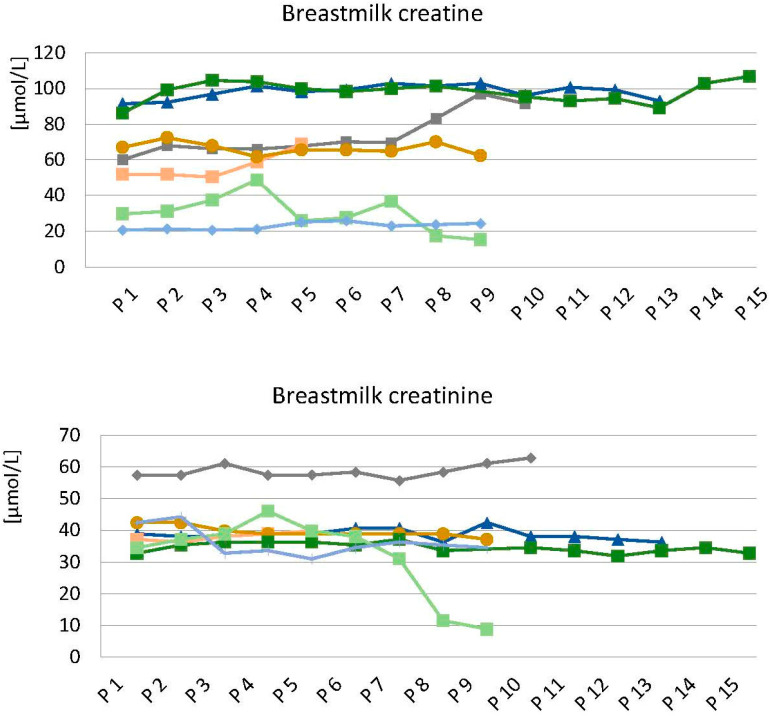
Time course of breast milk creatine and creatinine concentrations during a single breastfeeding session in seven mothers. Five to fifteen samples (P1 to P15) were obtained from each mother. Each color refers to the breast milk of an individual mother. Data are given in µmol/L.

The time course of breast milk creatine and creatinine concentrations during a single breastfeeding session in seven mothers is given in Figure 2. The mean intraindividual variability in breast milk Cr and Crn within one breastfeed was quite low (13 ± 11% and 11% ± 14%). The interindividual variabilities were considerably larger for Cr than for Crn (48% vs. 22% respectively) (Figure 2). In one participant, whole macronutrient analyses of all human milk samples, including fat, protein, lactose, and dry matter, were performed. The Cr concentration was neither related to Crn nor to any of the macronutrients (Table S1). For longitudinal HM Cr assessment, the HM samples were always drawn early within the first 5 min of a breastfeedi·ng session, after the initiation of milk injection.

### 3.3. Longitudinal Study on Breast Milk Creatine in Omnivore Mothers

Ten of the twelve participants were included in the evaluation. One mother was excluded for the early termination of breastfeeding and, in one mother, no colostrum sample could be obtained. In one participant, the week 2 sample was missing. All the samples were analyzed for Cr and Crn, whilst only 54 of 59 samples could be assessed for macronutrients due to insufficient sample volume.

**Figure 3:**
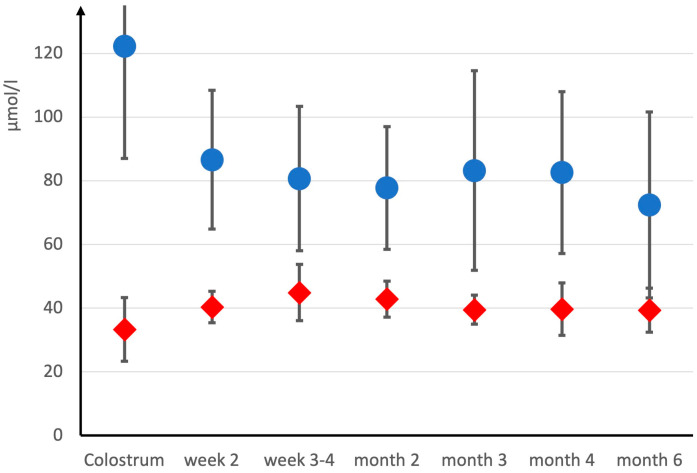
Human milk creatine (blue •) and creatinine (red •) across the first six months of breastfeeding. Data are given as mean ± SD.

**Figure 4:**
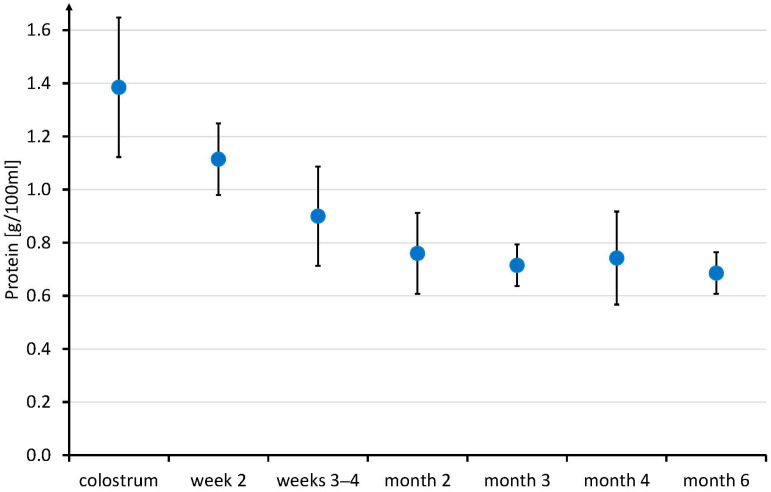
Human milk protein across the first six months of breastfeeding. Data are given as mean ± SD.

The Cr concentration was highest in the colostrum (Figure 3). It decreased from colostrum to mature breast milk across transition milk (*p* < 0.05), however, it did not change significantly thereafter. On average, the Cr concentration decreased by 34% within the first two weeks. In contrast, the mean breast milk Crn did not change significantly across the entire six months of lactation. Breast milk protein was highest in the colostrum and continuously decreased within the first 3 months of breastfeeding, as repeatedly reported [40] (Figure 4). The within-subject correlation between the creatine (mg/L) to protein (g/100 mL) ratio and the study timepoint was r(rmcorr) = 0.386, 95%-CI [0.157, 0.575], and thus, significantly differently from zero (*p* = 0.001). Therefore, the protein decrease was independent (significantly different) from the Cr decrease. (Figure 4) Fat and carbohydrates, on the other hand, did not change significantly within the study period (Table S2).

## 4. Discussion

### 4.1. Umbilical Creatine Flux

These are the first data on umbilical Cr flux in humans. The mean umbilical arterial and venous Cr levels were above the reported plasma Cr reference values during pregnancy (35.6 µM ± 15.15) [26]. Based on human placenta slices’ kinetics, maternal–fetal Cr transport has been hypothesized [41]. The Cr transporter (SLC6A8) has been shown to be expressed in human placental tissue, and term human placenta is also able to produce Cr and GAA [26,42]. However, currently, it is unknown whether there is maternal–fetal Cr transport at the end of pregnancy in vivo, which has been hypothesized in vitro [43]. No maternal–fetal Cr transfer has been found in sheep [44], in contrast to the rat [45], the spiny mouse, and rhesus macaque (S. Ellery, P. Grigsby, H. Dickinson, and D. Walker, unpublished observation [44]), where significant Cr transfer from mother to fetus was demonstrated [44]. In a recent human study, no difference between maternal and umbilical venous Cr was reported, however, the magnitude of the reported levels was far beyond the reported data [46].

Most importantly, the umbilical arterial Cr was higher than the umbilical vein Cr, suggesting the maturation of Cr syntheses in humans before term. Some of the fetal Cr (about 10%) seems to be lost in the placental circulation near term gestational age. It is unknown when Cr synthesis matures in fetal life. Rat embryos begin to express the Cr synthesis enzyme, guanidinoacetate methyltransferase (GAMT), in the last fifth of pregnancy [47]. This suggests that Cr synthesis occurs quite late in the fetus, although this study investigated enzyme levels only. Similarly, embryos of the spiny mouse, which, like the human, is a precocial mammal (giving birth to relatively developed, mature offspring), express renal and hepatic GAMT/AGAT enzymes at about 90% of gestation [24,48]. Rodent studies point towards the very late maturation of fetal Cr synthesis, and it would be of interest to know when Cr synthesis forms, matures, and becomes fully functional in the human fetus. Taking the umbilical flow into consideration, the present data suggest a negative fetal Cr flux of 2.07 mmol/kg/min at the end of pregnancy. The physiology behind this is unknown.

### 4.2. Intra- and Interindividual Breast Milk Creatine Variability

The composition of the aqueous phase of milk, as determined by the major osmotically active constituents, does not vary significantly within a feed (coefficient of variation < 13%) [49]. In our study, we found similar results for Cr and Crn (Figure 2). However, there was significant interindividual variability in Cr (48%). HM Cr was neither related to creatinine nor to any of the macronutrients (Table S1). Intraindividual variability may increase at the end of lactation (Figure 2). Therefore, with regard to the longitudinal analysis of HM Cr, it was decided to systematically use 10–15 mL of early-feed breast milk samples to balance the benefits of undisturbed breastfeeding against the methodological advantage of analyzing full breastfeeds.

### 4.3. Longitudinal Study on Breast Milk Creatine

The present data are the first systematic longitudinal data on breast milk Cr. Cr was always higher than Crn (which was always low), reconfirming previous observations and supporting the hypothesis of active Cr enrichment in HM [19,20,21,22]. Cr was highest in the colostrum and decreased thereafter, in contrast to Crn, which did not change significantly. It decreased by about one third (*p* < 0.05) from the colostrum (1st week) across the transition milk (2nd week) to mature breast milk (weeks 3–4) and did not change considerably thereafter (Figure 3). Cross-sectional data of the 80s [19] already supported such an hypothesis, whereas more recent data on pooled human milk samples did not find a difference between 1–2 or 5–6 weeks after birth [9]. The high Cr supply immediately after birth suggests a high need for this nutrient in the early neonatal period.

Cr secretion was independent from macronutrient secretion, supporting its importance. While HM fat and carbohydrate concentrations did not change significantly across the six months of breastfeeding (Table S2), protein decreased continuously (Figure 4) in contrast to Cr (Figure 3). The Cr (mg/L) to protein (g/100 mL) ratio significantly increased over time, suggesting the independent secretion of creatine and protein into HM.

As already discussed above, term newborns are able to synthesize Cr. It has been estimated that Cr neosynthesis places a significant metabolic burden on the infant, with approximately 27% of dietary glycine, 39% of arginine, and 75% of methionine being employed in Cr production [9]. It is well known that the metabolism of neonates is subjected to tremendous stress after birth, resulting in significant weight loss within the first 4 to 10 days [50,51]. Colostrum Cr may therefore reduce the metabolic burden of endogenous Cr synthesis, which, nevertheless, places a significant burden on the amino acid requirements of these infants [9]. This finding is consistent with the literature on Cr neosynthesis in other newborn mammals such as rat pups [23]. It would be of interest to study early colostrum in experimental animal models as well.

The present data suggest that there may be a critical Cr demand immediately after birth that needs to be covered by both neosynthesis and increased enteral supply via colostrum. Several preclinical studies have suggested that Cr is a promising neuroprotective intervention for hypoxic ischemic injury [27,28,29,30]. In vitro Cr plays a role in protecting cultured rat cardiomyocytes from hypoxic stress by enhancing the expression of hypoxia-inducible factor 1 (HIF-1), a master regulator of various anti-apoptotic mechanisms, as well as erythrocyte production [52]. Perinatal asphyxia is a leading cause of neonatal death. WHO estimates that a quarter of neonatal deaths, around 1 million annually, are caused directly by perinatal asphyxia [53]. Perinatal Cr metabolism may be directed to optimize natural neonatal neuroprotection given the threat of hypoxia.

## 5. Conclusions

In summary, the Cr flux in the umbilical cords of term infants is negative at the end of pregnancy, suggesting that fetal endogenous Cr synthesis near term meets the needs of these infants. Further research needs to show when exactly during pregnancy endogenous Cr synthesis matures. Especially in preterm infants, it is unknown whether endogenous Cr synthesis covers their needs. It has been hypothesized that peripheral and cerebral Cr levels decrease in preterm infants, leading to a relative systemic and cerebral Cr deficiency at term-corrected age. This reduction in Cr bioavailability may impair brain metabolism and development, ultimately leading to impaired neurodevelopmental outcomes [54].

With respect to HM, the intraindividual Cr variability during a single nursing session was low, suggesting that an early–mid sample was representative of the entire nursing session. HM Cr was not related to creatinine or any of the macronutrients. Across the first 6 months of breastfeeding, HM Cr was independent of Crn and macronutrient concentrations, suggesting that Cr is secreted independently into HM. Further research needs to analyze how Cr is secreted into HM. Finally, the high colostrum Cr suggests that there may be a critical time window of Cr requirement immediately after birth that needs to be covered by increased enteral intake. Since preclinical studies suggest that Cr is neuroprotective, further research is needed to investigate whether early Cr supplementation can improve outcomes in term infants and especially in preterm infants for whom colostrum is not available.

## Data Availability

Data are contained within the article and Supplementary Materials.

## Supplementary Materials

The following supporting information can be downloaded at: https://www.mdpi.com/article/10.3390/nu16030345/s1, Table S1: Human milk composition across a single breastfeeding session in one mother; Table S2: Breastmilk milk composition during the first six months of breastfeeding. Data is given as mean ± SD.
