# Supplementary material for: The Umbilical Cord Creatine Flux and Time Course of Human Milk Creatine across Lactation
Source: Nutrients. 2024 Jan 24;16(3):345. doi: 10.3390/nu16030345 (PMC10857059; doi:10.3390/nu16030345)
Supplement: Supplementary file 1 [file nutrients-16-00345-s001.zip › nutrients-2797915-supplementary.pdf]

Table S1. Human milk composition across a single breastfeeding session in one mother.

	Fat<br>g/100 ml	Proteine<br>g/100 ml	Lactose<br>g/100 ml	Creatine<br>µmol/l	Creatinine<br>µmol/l
Sample 1	0.45	0.8	7.5	60.2	57.5
Sample 2	-	-	-	67.9	57.5
Sample 3	1.5	0.8	7.4	66.3	61.1
Sample 4	2.6	0.8	7.35	66.2	57.5
Sample 5	2.8	0.75	7.5	67.9	57.5
Sample 6	2.5	0.9	7.5	70.2	58.4
Sample 7	3.55	0.7	7.6	68.6	57.8
Sample 8	5.35	0.6	7.3	83.3	58.4
Sample 9	5.5	0.7	7.4	97.3	61.1
Sample 10	-	-	-	91.6	62.8

Table S2. Breastmilk milk composition during the first six months of breastfeeding. Data is given as mean+/-SD.

	Protein g/100ml	Fat g/100ml	Carbohydrate g/100ml	Creatine µmol/l	Creatinine µmol/l
Colostrum	1,39 +/- 0,26	2,3 +/- 0,72	7,0 +/- 0,76	120 +/- 36	33 +/- 10
Week 2	1,01 +/- 0,24	1,9 +/- 0,76	7,0 +/- 0,50	89 +/- 23	40 +/- 5,0
Week 3-4	0,90 +/- 0,19	1,9 +/- 0,73	7,3 +/- 0,19	82 +/- 25	41 +/- 8,9
Month 2	0,76 +/- 0,15	2,3 +/- 0,92	7,4 +/- 0,69	79 +/- 20	43 +/- 5,6
Month 3	0,72 +/- 0,08	1,7 +/- 0,89	7,1 +/- 0,23	87 +/- 30	39 +/- 4,6
Month 4	0,74 +/- 0,18	1,9 +/- 1,02	7,1 +/- 0,20	86 +/- 23	40 +/- 8,2
Month 6	0,65 +/- 0,14	1,9 +/- 1,24	7,1 +/- 0,26	72 +/- 31	39 +/- 6,9
